# Experimental Physiology

**Published:** 1858-03

**Authors:** 


### EDITORIAL AND MISCELLANEOUS.

### Experimental Physiology.

*Ligation of the Heart of a Frog, for the purpose of demonstrating that Medicines, to become active, must necessarily pass through the Arterial system of Vessels.* By Prof. ALBERS, of Bonn.—(*Deutsche Klinik No.* 45.)—[Ed. translation.]

Nothing is easier than to pass a ligature around the base of the heart of a frog. A longitudinal incision passing through the thoracic walls exposes the pericardium, and if this be drawn aside and incised, the heart thus exposed may be surrounded at its base with a ligature. As soon as the circulation is arrested, the heart turns pale—emptying itself of the blood it contains. By degrees it ceases to beat, or if the pulsations continue, become very feeble. These phenomena strikingly resemble those produced by the irritation of the superior portion of the spinal cord by a current of electricity, or the repose of the heart produced by théine and caféine (the active principles of tea and coffee.) The animal, during the time that the heart is ligated, loses none of its activity, conducting itself as before the operation. Frequent respiratory movements are observed if the pleura has not been wounded.

If, at the expiration of a quarter or half an hour, or even an hour or more, the ligature is removed, (which should have been drawn sufficiently tight to have wounded the structure of the organ) the heart resumes its natural color, and as soon as it is filled with blood, commences pulsating. During the arrest of the circulation by the ligation of the heart, the most active medicines introduced into the stomach, or a wound, produce no effect; but, as soon as the ligature is removed, their action commences. It is a remarkable fact, that substances, which, under ordinary circumstances, will not manifest themselves in less time than from three to twelve minutes, will produce an evident effect in from one eighth to a half minute after the ligature is removed when the heart has been ligated half an hour, two hours, or even three hours or more. We can but conclude, from this fact, that the ligation of the heart does not by any means prevent absorption; that substances introduced into the organism readily pass into the veins and lymphatics, notwithstanding the arrest of the circulation, but to become active must pass through the heart and arteries into the capillary system of vessels. Besides, as to the promptitude with which various substances act, there is a marked difference in the same substances upon animals which have not been submitted to the operation above alluded to.

The author has experimented with *digitaline*, *strychnine*, *caféine* and *conicine*, and has observed the same facts.

Thus, for example, a quarter of a grain of the nitrate of strychnine was placed under the skin of a frog, the heart having been previously ligated; at the expiration of three quarters of an hour the poison had produced no effect. The ligature was then removed, and in a quarter of a minute the strychnine had attained all its energy, producing the death of the frog.

The ligation of the heart is greatly preferable to the excision of this organ, when we wish to demonstrate the influence of the circulation upon the activity of medicines or poisons, as the latter operation gives place to hemorrhages which so exhaust the animal that many of the functions are greatly enfeebled or completely suspended.

